# Left Atrial Fibrosis after Single Shot Guided Pulmonary Vein Isolation

**DOI:** 10.3390/jcm10194478

**Published:** 2021-09-28

**Authors:** Shibu Mathew, Islam Saboukh, Parminder Singh, Bastian Fries, Victoria Johnson, Nikita Schneider, Christian Fraebel, Ritvan Chasan, Christian W. Hamm, Jörn Schmitt

**Affiliations:** Department of Cardiology, University Hospital of Giessen, 35392 Giessen, Germany; eslam_abdelgwad@med.kfs.edu.eg (I.S.); sikka.parminder@gmail.com (P.S.); bastian.fries@outlook.com (B.F.); johnson.victoria.louise@gmail.com (V.J.); nikitaschneider@t-online.de (N.S.); christian.fraebel@live.de (C.F.); r-chasan@hotmail.com (R.C.); christian.hamm@innere.med.uni-giessen.de (C.W.H.); joern.schmitt@innere.med.uni-giessen.de (J.S.)

**Keywords:** cryoballoon, atrial fibrillation, pulmonary vein isolation, posterior wall, catheter ablation, fibrosis

## Abstract

Cryoballoon (CB)-based pulmonary vein isolation (PVI) is an effective treatment modality for patients with atrial fibrillation (AF) with encouraging acute and long-term outcome data. However, the size of collaterally created lesion sets adjacent to the pulmonary veins (PVs) remains unclear, especially when CB ablation is performed with individualized time-to-isolation (TTI) protocols. This study seeks to investigate the extension of lesions at the posterior wall and the roof of the left atrium (LA). Thirty patients with paroxysmal or persistent AF underwent ablation with a fourth-generation CB. The individual freeze-cycle duration was set at TTI + 120 s. A total of 120 PVs were identified, and all were successfully isolated. A three-dimensional electroanatomical high-density (HD) mapping of the LA was performed in every patient before and after PVI. The surface areas of the posterior wall and LA roof were measured and compared with lesion extension after PVI. After CB ablation, 65.6 ± 16.9% of the posterior wall and 75.4 ± 18.4% of the LA roof remained unablated. In addition, non-antral lesion formation was observed in every patient in at least one PV. After CB ablation, anterior antral parts of the superior PVs showed the greatest unablated areas compared with the other antral areas. HD re-mapping after CB-based PVI demonstrated that major regions of the posterior wall and roof remained electrically normal and unaffected. Unablated antral areas were localized predominantly in the anterior segments of the superior PVs and may be partly responsible for AF recurrence.

## 1. Introduction

Pulmonary vein isolation (PVI) is an established treatment modality in patients with paroxysmal atrial fibrillation (PAF) and persistent atrial fibrillation (PersAF) [1,2,3,4]. Several studies have demonstrated high acute success rates and non-inferior outcomes for PVI when using cryoballoon (CB) ablation compared to radiofrequency catheter ablation [5,6]. These results are primarily based on the high durability of implemented lesion sets [7,8]. Different ablation strategies have been proposed in recent years and are mostly characterized by a fixed freeze-cycle duration of 180 to 240 s. A recent randomized trial demonstrated a comparable clinical outcome after 120 and 240 s freeze-cycle durations [6]. Additionally, individualized shorter freeze-cycle durations were recently proposed as an alternative ablation strategy. The main concept is here to apply the time-to-isolation (TTI) protocol: one freeze application with a predefined freeze time after isolation of the PV [9,10]. However, the size of collaterally created lesions adjacent to the pulmonary veins (PVs) after TTI-derived CB ablation are not clear. Due to the spherical nature of the CB, proximal balloon areas can be brought into contact with atrial tissue and create wide antral lesion sets around the PVs, especially at the posterior left atrial (LA) wall. This has been investigated in a few studies in the acute phase after CB PVI with 3D electroanatomical LA mapping [11,12,13]. However, all of these studies were performed without high-density (HD) mapping, with previous generations of the CB (first- and second-generation CB) in small cohorts of patients, and with fixed dosing protocols of cryofreezes. This study seeks to investigate the extension of low-voltage zones (LVZs) after PVI with a fourth-generation CB (CB4) based on individualized TTI protocols.

## 2. Materials and Methods

### 2.1. Study Population

The patient population comprised 30 patients with symptomatic PAF or PersAF (duration ≤ 6 months). Exclusion criteria were prior left atrial (LA) ablation attempts, LA diameter > 60 mm, severe valvular heart disease, or contraindications to postinterventional oral anticoagulation. In cases where oral anticoagulation was insufficient, transesophageal echocardiography was performed prior to PVI to rule out intracardiac thrombi and to assess the LA diameter. No further pre-procedural imaging was performed. All patients gave written informed consent for the use of their data, and all patient data were anonymized. This study was approved by the local Ethics Review Board (protocol number: AZ 199/15), according to the principles of the Declaration of Helsinki 2014.

### 2.2. Electrophysiological Study and Electroanatomical Mapping

#### 2.2.1. Intraprocedural Management

The procedure management was previously described in [2]. In brief, the procedure was performed under deep sedation using midazolam, sufentanil, and propofol. One 6F diagnostic catheter was positioned within the coronary sinus and a single transseptal puncture was performed under fluoroscopic guidance. Selective PV angiography was performed to identify the PV ostia. The transseptal sheath was exchanged for a 12F steerable sheath (Flexcath Advance, Medtronic Inc., Minneapolis, MN, USA). After transseptal puncture, heparin boluses were administered to achieve an activated clotting time of >300 s. 

#### 2.2.2. Electroanatomical Mapping before PVI 

**Figure 1:**
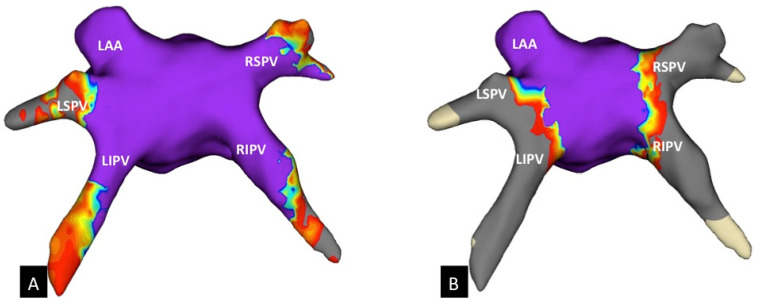
3D-HD map of the left atrium. (**A**) The initial map before cryoballoon ablation in a superior view. (**B**) Lesion extension after CB-based PVI in a superior view. Note the unablated area at the roof and the posterior wall between the PVs and the antrum of the LSPV. 3D-HD, high-density; RSPV, right superior pulmonary vein; RIPV, right inferior pulmonary vein; LSPV, left superior pulmonary vein; LIPV, left inferior pulmonary vein; LAA, left atrial appendage.

Before performing the CB procedure, a 3D bipolar voltage map of the LA was obtained utilizing a diagnostic high-density (HD) catheter (Advisor HD Grid, Abbott Laboratories, Minneapolis, MN, USA) and the Ensite Precision System (Abbott Laboratories, Minneapolis, MN, USA) in every patient (Figure 1A). All 3D-HD maps were obtained in sinus rhythm. In patients with AF at baseline, electrical cardioversion was performed prior to LA mapping. Low-voltage amplitudes/fibrosis and a scar in bipolar electrograms were defined as <0.5 mV and <0.1 mV, respectively [14]. 

#### 2.2.3. PVI Using the CB4

The CB-based procedure was previously described in [2]. The 28 mm CB4 (Medtronic Inc., Minneapolis, MN, USA) and a spiral mapping catheter (20 mm diameter; Achieve, Medtronic Inc., Minneapolis, MN, USA) were introduced into the LA via the 12F steerable sheath. To achieve PV occlusion, the CB was inflated proximal to the PV ostium. Complete occlusion of the PV ostium was verified by injections of contrast media. The standard freeze-cycle duration was TTI + 120 s [9]. In cases without PV live recordings, a conventional freeze-cycle duration of 180 s was performed. No additional bonus freeze cycle was applied. During PVI of the septal PVs, continuous phrenic nerve (PN) pacing was performed using a diagnostic catheter placed within the superior vena cava. Pacing was set at maximum output and pulse width. PN capture was monitored by the tactile feedback of diaphragmatic contraction. The right diaphragm compound motor activation potential (CMAP) was also assessed. In cases of persistent PN palsy, no further cryoenergy was delivered along the septal PVs [15].

#### 2.2.4. Electroanatomical Mapping after PVI

After PVI, 3D-HD mapping of the LA was repeated (Figure 1B). All maps were performed during sinus rhythm with settings for bipolar low-voltage detection, as described above. 

#### 2.2.5. LVZ Analysis before and after CB Ablation

**Figure 2:**
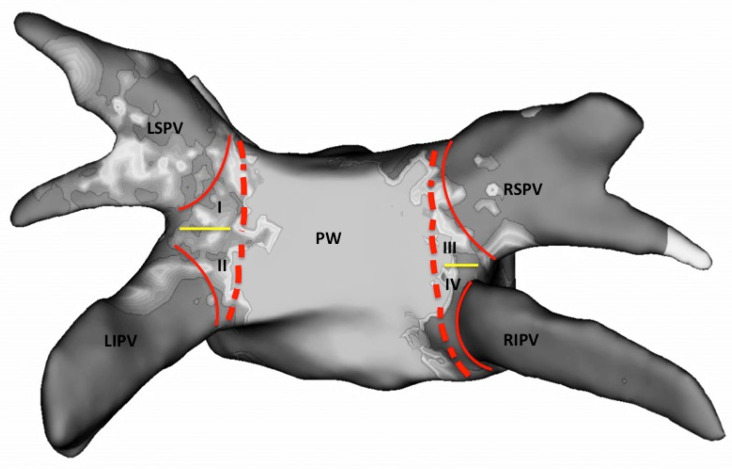
3D map in a posterior–anterior view (PA) with identification of PV ostia and antral area of the pulmonary veins. Red lines mark the PV ostia. Red dotted lines mark the antral area of the PVs (I = antrum LSPV; II = antrum LIPV; III = antrum RSPV; IV = antrum RIPV). Yellow lines mark the carina between the pulmonary veins. RSPV, right superior pulmonary vein; RIPV, right inferior pulmonary vein; LSPV, left superior pulmonary vein; LIPV, left inferior pulmonary vein; PW, posterior wall.

**Figure 3:**
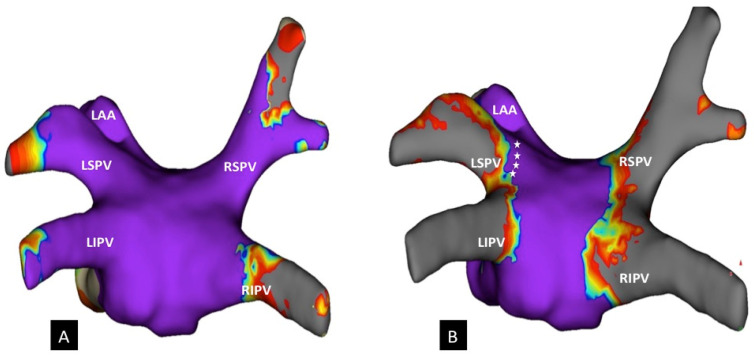
3D-HD map of the left atrium in a posterior–anterior view (PA). (**A**) The initial map before cryoballoon ablation. (**B**) Lesion extension after CB-based PVI in a posterior–anterior view. The white asterisks mark an area with preserved bipolar voltage at the posterior superior antrum of the LSPV. 3D-HD, high-density; RSPV, right superior pulmonary vein; RIPV, right inferior pulmonary vein; LSPV, left superior pulmonary vein; LIPV, left inferior pulmonary vein; LAA, left atrial appendage.

For standardized analysis, the PV ostia were defined as the point of maximum inflection between the PV wall and the LA wall. Surface areas of the posterior wall (between the PVs) and LA roof (between the superior ostia of the PVs) were measured manually with the area measurement function of the 3D mapping system before CB ablation (Figure 2). After ablation, the surface area of unablated regions with preserved bipolar electrogram amplitudes (amplitude > 0.5 mV) within the posterior wall and roof were also measured. In addition, if preserved bipolar electrogram amplitudes along the antral area of the PVs were present, the unablated surface area in every PV was measured (Figure 3). 

### 2.3. Postprocedural Care

After ablation, transthoracic echocardiography was performed in every patient to exclude pericardial effusion. Low-molecular-weight heparin was administered in patients on vitamin K antagonists and an INR < 2.0 until a therapeutic INR of 2–3 was achieved. In patients treated with direct oral anticoagulants (DOACs), anticoagulation was resumed 6 h after ablation. Anticoagulation was continued for at least 3 months and afterwards based on their individual CHA_2_DS_2_-VASc score. All patients were treated with proton-pump inhibitors for 6 weeks.

### 2.4. Statistical Analysis

Continuous variables were summarized as mean ± standard deviation or median (interquartile range (IQR)). Categorical variables were expressed as numbers and frequencies (percentages). 

## 3. Results

### 3.1. Patient Characteristics and Intraprocedural Results

Baseline patient characteristics are depicted in Table 1. A total of 120 PVs were treated in 30 patients (Table 2). All PVs were isolated exclusively with the 28 mm CB4. No additional lesions with radiofrequency energy were applied. TTI was achieved in 80/120 (67%) PVs. The mean TTI was 51.8 ± 15.8 s for the LSPV and 38.2 ± 20.1 s, 43.8 ± 25.3 s, and 35.4 ± 14.1 s for the LIPV, RIPV, and RSPV, respectively (Table 3). The remaining PVs without online registration of PV signal disappearance during the freeze cycle were isolated with a standard protocol of 180 s. No severe complications occurred in this patient population. An intermittent phrenic nerve palsy occurred in one patient, which resolved during the procedure. 

### 3.2. Electroanatomical Features

High-resolution mapping of the LA and the PVs was performed in all patients before and after PVI. The mean number of LA points acquired by the multielectrode catheter was 2556 ± 495. The mean surface area of the posterior wall before CB ablation was 25.5 ± 7.6 cm^2^ (Table 4). After CB ablation, the unablated surface area of the posterior wall with preserved bipolar electrograms was 16.6 ± 5.8 cm^2^ (65.6 ± 16.9%), whereas 75.4 ± 18.4% of the LA roof remained electrically intact. In three patients (10%) the entire LA roof remained unablated. 

**Figure 4:**
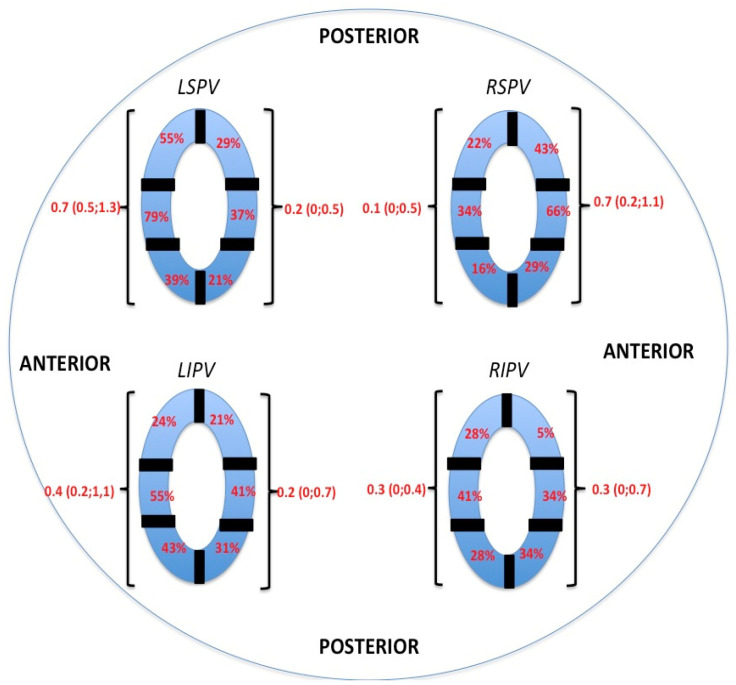
Analysis of areas with preserved antral electrical signals after successful PVI. Each pulmonary vein is divided into six segments showing the distribution of regions with preserved local electrogram amplitudes. Continuous variables are summarized as median and interquartile range (IQR).

Quantitatively, non-antral lesion formation was observed in at least one PV in all 30 patients (100%). Qualitatively, the locations and sizes of unablated areas (antrum to ablation lesion) differed among the patients. The largest areas of intact and antrally located electrograms after successfully performed PVI were displayed at the antral parts of the superior PVs (LSPV: 0.7 cm^2^ (IQR: 0.5; 1.3); RSPV: 0.7 cm^2^ (I\QR:0.2; 1.1)). This was more distinct in the most superior regions of the LSPV and RSPV. For inferior PVs, preserved electrogram amplitudes were more evident in medial to inferior areas, especially at the midsection of the anterior inferior ridge of the LIPV (Figure 4).

## 4. Discussion

To our knowledge, this is the first report of the use of a standardized TTI protocol that assesses the acute isolation area after CB4-based PVI with 3D-HD maps. The main findings of this study are that (1) after successful CB ablation 65.6 ± 16.9% of the posterior wall and 75.4 ± 18.4% of the LA roof remained unablated, and (2) the anterior antral regions of the right and left superior pulmonary veins displayed the greatest areas of preserved electrograms.

### 4.1. Electroanatomical Features: Extent of Lesion Formation Adjacent to PVs

Only a few studies have analyzed the extent of the isolated area after CB ablation using 3D mapping systems in patients with PAF during the acute phase. Chierchia et al. analyzed eight patients treated with a first-generation cryoballoon (23 or 28 mm balloon) and two freeze cycles of 300 s duration [13]. Reddy et al. also examined eight patients who underwent the procedure with a 28 mm CB and a 240 s freeze duration [12]. In 2015, Kenigsberg et al. reported results from forty-three patients in whom PVI was performed with the second-generation CB and two 180 s freeze cycles [11]. The major difference between the present study and previously published studies is that the CB4 (28 mm) was used exclusively with an individualized TTI protocol. In addition, 3D-HD mapping of the LA was performed in all patients before and after the procedure. It is well known that accuracy and more detailed voltage maps may depend on different variables, such as interelectrode spacing, electrode size, and the cumulative number of acquired electrical points [14]. All previously published studies concerning acute-phase fibrosis after CB ablation were performed without HD LA mapping and with partly different settings of bipolar amplitudes, which no longer represent the state of the art in low-voltage detection [11]. Therefore, a detailed comparison of these findings with our data is difficult. As mentioned above, we also saw areas of isolated structures adjacent to the PVs, including the posterior wall and the roof of the LA. However, the unablated area of the posterior wall was much greater in our study cohort than in previously published studies. Kenigsberg et al. reported that 27% of the posterior wall was unablated after CB ablation, whereas in our cohort the unablated area was 65.6 ± 16.9% [11]. 

There are a number of potential reasons for these conflicting results on smaller lesion extension to the posterior wall. The differences could have a technical basis due to the different settings of the electroanatomical bipolar voltage maps, as mentioned above. In addition, differences in the patient populations might influence these results since we included patients with PersAF and PAF. PV size and balloon position may also play a role in collaterally ablated areas near the PVs. Greater PV diameter should result in more distal ablation lesions and fewer LVZs within the posterior wall. The use of an individualized TTI protocol was also a major difference compared with previous studies. The clinical benefit of shorter freeze-cycle durations is still under debate; however, preclinical and clinical randomized studies have already shown that shorter freeze-cycle durations (120 s vs. 240 s) result in similar outcome data [6,16]. Freezes of shorter duration may translate into smaller lesion sets in areas adjacent to the PVs. Recently, Miyazaki et al. analyzed the extent of fibrosis after CB PVI during the chronic phase in re-ablation procedures [17]. During their initial CB procedure, the freeze cycle was 180 s without bonus application. They also observed areas of fibrosis within the posterior wall, which were also smaller than in the previously published data on the acute phase [17]. Our findings are consistent with these data on LVZs during the chronic phase since the sizes of our isolated areas were similar and the freeze cycles shorter than in previously published studies. The use of the CB4 most likely did not play a key role in this setting, since technical changes compared with previous balloon technology are minor [18,19]. 

The clinical impact of these observations of collaterally ablated parts of the LA needs to be analyzed in a larger patient population, especially the question of whether these lesions lead to iatrogenic atrial tachycardias. It has been recently reported by Gunarwardene et al. that patients may present with atrial tachycardias after CB ablation due to residual fractionated signals along the previous cryothermal lesions [20]. 

### 4.2. Electroanatomical Features: Extent of Lesion Formation at the PV Ostia

The need for wide circumferential ablation lines when performing complete PVI has been well investigated [21,22]. Interestingly, in every patient in our population we observed a region of non-antral lesion formation in at least one PV, even though all PVs were acutely isolated during the procedure. This was more evident for the antral and anterior regions of the superior PVs. Several studies analyzed the durability and predilection sites of gaps in radiofrequency re-do procedures performed after procedures that were initially conducted using CB ablation [23,24,25]. Our results are in line with these studies, where typical sites of reconduction were at the anterior-superior regions of the LSPV or the RSPV or the most inferior parts of the inferior PVs. Different factors might influence these sites of reconduction, including an interaction of anatomical circumstances and energy delivery. Recently, Chen et al. reported in the ICE Re-Map study that lesions around the LPV were more durable after freezes of 240 s than after 180 s freezes [26]. Thicker myocardial tissue along the anterior ridge of the LPV and the LAA may require additional freeze time for transmural and durable PVI. Another typical region of reconduction is the inferior part of the inferior PVs. To reach optimal temperatures, many operators perform pull-down maneuvers after at least 60 s. This shorter duration of freezes might also result in less-durable lesions with fewer LVZs in these areas, even though the PVs show acute isolation. However, whether these antral areas of preserved bipolar voltage amplitudes lead to a recurrence of AF needs to be re-evaluated in further studies.

## 5. Conclusions

High-density re-mapping after CB-based PVI demonstrated that major portions of the atrial posterior wall and roof remained electrically normal and unaffected. Unablated antral areas were predominantly in the anterior parts of the superior PVs, which may translate into a recurrence of AF.

## Figures and Tables

**Table 1:** Baseline characteristics and medication before catheter ablation. Values are expressed as *n* (%) or mean ± standard deviation. AF, atrial fibrillation; LA, left atrium; LVEF, left ventricular ejection fraction.

Variable	
Patients, *n*	30
Age (years)	64 ± 10
Female gender, *n* (%)	10 (30)
Paroxysmal AF, *n* (%)	14 (46.7)
Persistent AF, *n* (%)	16 (53.3)
LA size (mm)	42.7 ± 4.5
LVEF (%)	55.8 ± 7.8
Arterial hypertension, *n* (%)	23 (76.7)
Diabetes mellitus, *n* (%)	6 (20)
Coronary artery disease, *n* (%)	3 (10)
Medication at baseline	
• B-Blocker, *n* (%)	12 (40)
• Class Ic, *n* (%)	2 (6.7)
• Class III, *n* (%)	2 (6.7)
CHA_2_DS_2_-VASc-Score	2.5 ± 1.4

**Table 2:** Procedural details. Values are expressed as *n* (%) or mean ± standard deviation. PV(s) = pulmonary vein(s), PVI = pulmonary vein isolation, temp. = temperature, and TTI = time-to-isolation.

Variable	
Number of PVs, *n*	120
Number of isolated PVs, *n* (%)	120/120 (100)
Mean overall minimum temp. (°C)	−49.9 ± 5.4
Mean overall time to PVI (s)	42.7 ± 19.4
Mean overall temp at TTI (°C)	−35.2 ± 8.8
Mean duration of total freezing time (s)	167.2 ± 27.8

**Table 3:** Procedural details—individual pulmonary veins. Values are expressed as *n* (%) or mean ± standard deviation. PVI, pulmonary vein isolation; RSPV, right superior pulmonary vein; RIPV, right inferior pulmonary vein; LSPV, left superior pulmonary vein; LIPV, left inferior pulmonary vein; LCPV, left common pulmonary vein; temp., temperature; TTI, time-to-isolation.

Variable	
RSPV	
Mean minimum temp. (°C)	−53 ± 5.9
Duration of total freezing time (s)	149.3 ± 28.8
Time to PVI, (s)	35.4 ± 14.1
Mean temp at TTI (°C)	−34.3 ± 8.9
RIPV	
Mean minimum temp. (°C)	−48.7 ± 4.8
Duration of total freezing time (s)	168.4 ± 20.4
Time to PVI (s)	43.8 ± 25.3
Mean temp at TTI (°C)	−34.3 ± 10.6
LSPV	
Mean minimum temp. (°C)	−50.9 ± 4.7
Duration of total freezing time (s)	173.2 ± 15
Time to PVI (s)	51.8 ± 15.8
Mean temp at TTI (°C)	−39.1 ± 6.3
LIPV	
Mean minimum temp. (°C)	−47 ± 4.3
Duration of total freezing time (s)	167.5 ± 20.5
Time to PVI (s)	38.2 ± 20.1
Mean temp at TTI (°C)	−31.3 ± 8.6

**Table 4:** Procedural details—low-voltage zones (LVZs) after cryoballoon (CB) ablation. Values expressed as *n* (%) or mean ± standard deviation. CB, cryoballoon; LA, left atrium; PV, pulmonary vein.

Variable	
***Posterior wall* (*between PVs*)**	
Area before CB ablation (cm^2^)	25.5 ± 7.6
Area unablated (cm^2^)	16.6 ± 5.8
Area unablated (%)	65.6 ± 16.9
***LA Roof***	
Area before CB Ablation (cm^2^)	6.0 ± 1.7
Area unablated (cm^2^)	4.5 ± 1.8
Area unablated (%)	75.4 ± 18.4

## Data Availability

The data presented in this study are available on request from the corresponding author.
